# Unravelling a simple method for the low temperature synthesis of silicon nanocrystals and monolithic nanocrystalline thin films

**DOI:** 10.1038/srep40553

**Published:** 2017-01-16

**Authors:** Ka-Hyun Kim, Erik V. Johnson, Andrey G. Kazanskii, Mark V. Khenkin, Pere Roca i Cabarrocas

**Affiliations:** 1 KIER-UNIST Advanced Center for Energy, Korea Institute of Energy Research, 44919, Ulsan, South Korea; 2 LPICM, CNRS, Ecole Polytechnique, Université Paris-Saclay, 91128 Palaiseau, France; 3 TOTAL New Energies, 92069 Paris, France; 4 Faculty of Physics, Moscow State University, Moscow, 119991, Russia

## Abstract

In this work, we present new results on the plasma processing and structure of hydrogenated polymorphous silicon (pm-Si:H) thin films. pm-Si:H thin films consist of a low volume fraction of silicon nanocrystals embedded in a silicon matrix with medium range order, and they possess this morphology as a significant contribution to their growth comes from the impact on the substrate of silicon clusters and nanocrystals synthesized in the plasma. Quadrupole mass spectrometry, ion flux measurements, and material characterization by transmission electron microscopy (TEM) and atomic force microscopy all provide insight on the contribution to the growth by silicon nanocrystals during PECVD deposition. In particular, cross-section TEM measurements show for the first time that the silicon nanocrystals are uniformly distributed across the thickness of the pm-Si:H film. Moreover, parametric studies indicate that the best pm-Si:H material is obtained at the conditions after the transition between a pristine plasma and one containing nanocrystals, namely a total gas pressure around 2 Torr and a silane to hydrogen ratio between 0.05 to 0.1. From a practical point of view these conditions also correspond to the highest deposition rate achievable for a given RF power and silane flow rate.

Nanocrystals, having a dimension on the order of a few nanometers, exhibit quantum effects that are useful in applications such as memory devices, solar cells, thermoelectrics, light emitting diodes, spintronic devices and printable electronics^1^,^2^,^3^,^4^,^5^,^6^,^7^. Solid-phase synthesis of silicon nanocrystals can be done by sputtering films of silicon oxide, nitride, or silicon carbide alloys, and subjecting them to a high temperature annealing to nucleate and grow randomly dispersed silicon nanocrystals^8^. This is also a popular method to fabricate superlattices of silicon nanocrystals if alternating layers of silicon oxide and silicon-rich oxide are sequentially deposited^9^,^10^. However, the high temperature of these processes (around 800 °C–1100 °C) is an obstacle to achieving low cost fabrication and mass production. In order to lower the process temperature, gas-phase synthesis of silicon nanocrystals can be used, where the gas precursor is dissociated by plasma or laser^11^,^12^,^13^,^14^,^15^. Over the past decade, we have performed detailed studies on the growth, optoelectronic properties and the application of hydrogenated polymorphous silicon (pm-Si:H) thin films to solar cells^16^,^17^,^18^. Indeed, the optoelectronic properties of pm-Si:H are outstanding and allow us to envision single junction PIN solar cells with stable efficiencies of 12%^19^. However, the growth process of this nanostructured material is still a matter of discussion, as it can be confused with hydrogenated protocrystalline silicon (pc-Si:H), grown under conditions that for higher thicknesses result in microcrystalline material^20^,^21^. Indeed, both materials are grown from highly hydrogen diluted silane plasmas, and they are both depicted as highly heterogeneous mixed-phase materials at scales close to tens of nanometers. Due to the mixed-phase nature of these materials, their optical band gap is wider than that of a-Si:H^16^,^22^, and both materials show a characteristic low temperature peak near 400 °C in the hydrogen exodiffusion spectra^23^,^24^. However, distinct differences still exist in the plasma physics during their deposition and their material nanostructures^25^. In particular, while the growth of pc-Si:H is assumed to rely on SiH_3_ radicals, pm-Si:H deposition relies on the contribution of plasma synthesized nanocrystals^21^,^25^. Due to the growth mechanism of pc-Si:H, its mixed-phase nanostructure appears after an amorphous incubation layer. In other words, the heterogeneous nature of pc-Si:H only appears after some thickness. On the contrary, the mixed-phase nature of pm-Si:H has been assumed to be homogeneous through the film thickness, presumably because of the different deposition mechanism. This is an important point for practical applications because the nanostructure and physical properties of pc-Si:H mainly depend on both hydrogen dilution and film thickness while those of pm-Si:H are independent of thickness.

In this work, we focus on this nanocrystal-based growth process and for the first time provide experimental evidence via Transmission Electron Microscopy (TEM) of the homogeneous distribution of silicon nanocrystals through the thickness of the layer. Moreover, the correlation between the plasma conditions (in particular the ion flux) and the structure of the deposited material is carefully examined by real time plasma monitoring and characterization of thin film material properties.

## Results and Discussion

**Figure 1:**
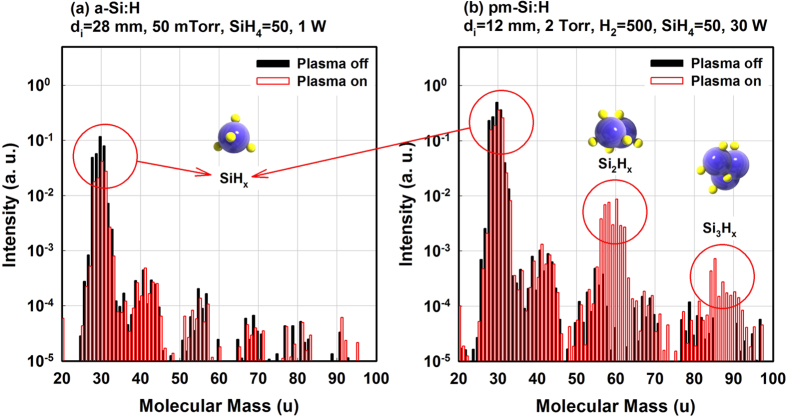
QMS scanned molecular mass distribution for (**a**) a-Si:H and (**b**) pm-Si:H deposition conditions at plasma on/off condition. Note that in pm-Si:H deposition conditions, higher-order silane species such as Si_2_H_x_ and Si_3_H_x_ are detected.

Figure 1 shows quadrupole mass spectrometry (QMS) scans giving the molecular mass distribution of the species detected in the pumping line under conditions of a-Si:H and pm-Si:H deposition. For both the a-Si:H and pm-Si:H process conditions, there is a decrease in the SiH_x_ signal (molecular mass ~30), which is attributed to the fact that the silane is dissociated and consumed by the deposition. Any increase of SiH_x_ species is undetectable because the QMS was installed at the gas exhaust line, between the throttle valve and the dry roots pump. Radicals are assumed to contribute to deposition before reaching the QMS. An interesting point is that poly-silane species, such as Si_2_H_x_ (molecular mass ~60) and Si_3_H_x_ (molecular mass ~90), are significantly detected during pm-Si:H deposition while they are absent under a-Si:H deposition conditions. The detection of the higher order silane species (precursors for the formation of nanocrystals) is one of the features that best distinguishes the pm-Si:H growth process with respect to that of a-Si:H.

The first parameter we examine is the effect of relative gas flow rates. The dilution of silane in hydrogen strongly affects the plasma processes, and in particular those processes leading to cluster formation. The gas flow ratio of SiH_4_ and H_2_ was varied while the other process parameters were kept constant: pressure = 2.0 Torr, T_s_ = 175 °C and RF Power = 30 W. The gas flow ratio R is defined as;


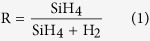


where SiH_4_ and H_2_ are the gas flow rates in sccm.

**Figure 2:**
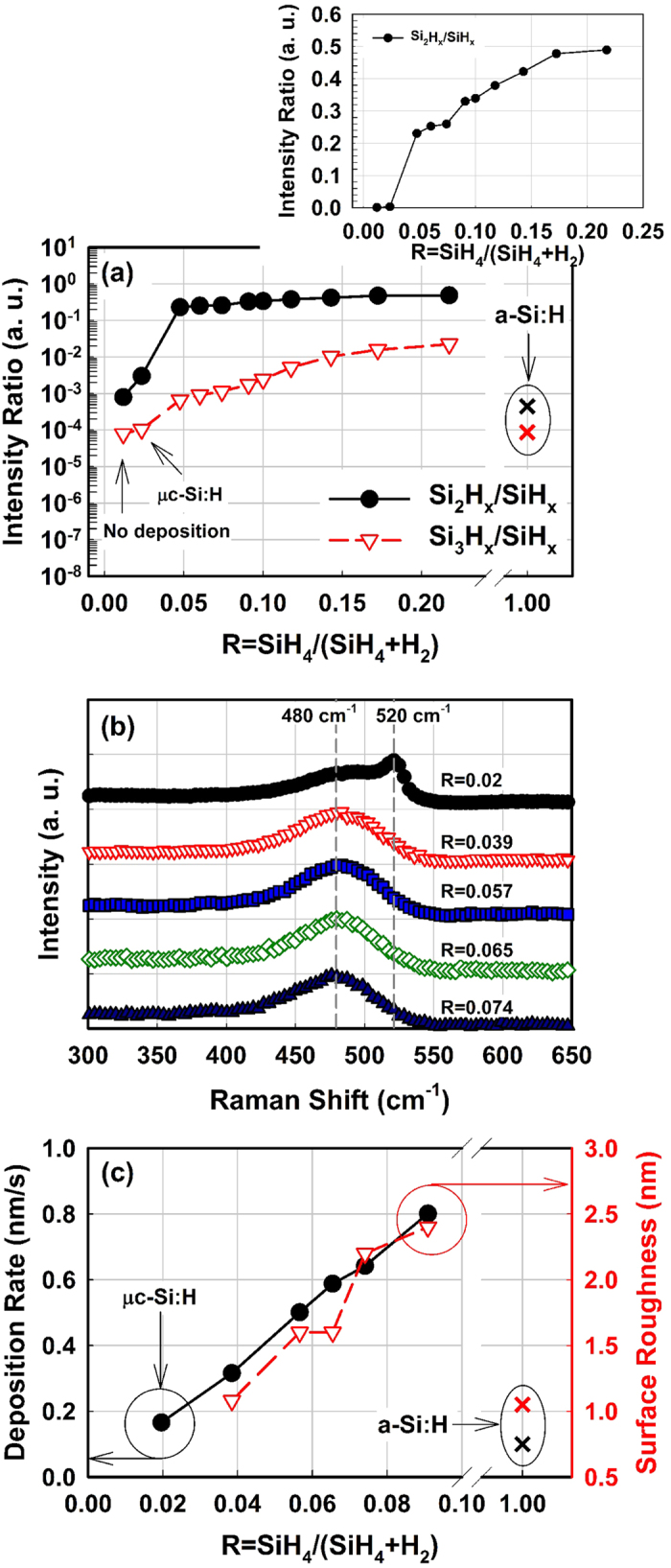
Representative results of gas flow series. (**a**) QMS analysis of signal intensity ratio of Si_2_H_x_ to SiH_x_ and Si_3_H_x_ to SiH_x_ as functions of gas flow ratio R. (**b**) Raman spectra of layers deposited at various gas flow ratio R. A transition from amorphous (480 cm^−1^) to crystalline (520 cm^−1^) is notably seen at R = 0.02. (**c**) deposition rate and surface roughness as functions of SiH_4_ flow. Inset in (**a**) presents the same results in a linear scale.

Two QMS signal intensity ratios (Si_2_H_x_/SiH_x_ and Si_3_Hx/SiH_x_) are shown in Fig. 2(a) as functions of the gas flow ratio R. The ratios for a-Si:H deposition (45 mTorr, 1 W, pure silane dissociation) appear as crosses in Fig. 2. For the dilution series, total pressure and RF power were fixed at 2 Torr and 30 W, respectively. The total gas pressure was kept constant by adjusting the throttle valve. As expected from modelling, increasing the H_2_ dilution reduces the Si_2_H_x_ and Si_3_H_x_ concentration in the plasma^26^. Furthermore, as indicated on Fig. 2(a), the nature of the film deposition is strongly correlated with the Si_2_H_x_/SiH_x_ ratio. For example, there is no deposition on glass substrates at a gas flow ratio R of 0.01, when the Si_2_H_x_ signal intensity ratio is small (~10^−3^). The deposited material is μc-Si:H at R = 0.02 when the ratio is ~3 × 10^−3^. Above a gas flow ratio R of 0.05, the Si_2_H_x_/SiH_x_ signal intensity ratio increases by almost two orders of magnitude (>10^−1^) and the plasma goes into the regime of pm-Si:H deposition.

The change in SiH_4_ to H_2_ gas flow ratio R results into a notable transition from amorphous to μc-Si:H to the film growth. Figure 2(b) shows Raman spectra of films deposited at various gas flow ratio R. In the Raman spectra, a transition from amorphous (480 cm^−1^) to crystalline (520 cm^−1^) is notably seen at R = 0.02. At high gas flow ratio R over 0.039, Raman spectra only show single broad peak centered at 480 cm^−1^, which indicates transverse optical (TO) phonon mode of amorphous silicon. At gas flow ratio R of 0.02, crystalline silicon peak is detected at 520 cm^−1^ as well as TO mode of a-Si:H at 480 cm^−1^, which is typical evidence of μc-Si:H growth. Roles of hydrogen for the growth of μc-Si:H are well discussed and classified into three categories: etching, chemical annealing and surface diffusion^27^. Usually the three models are discussed and considered to the growing film surface, but our result also suggests that effect of hydrogen dilution works not only to the growing film surface, but also to the surface of plasma synthesized nanocrystals.

The result of Fig. 2(a) also makes an interesting point on the mechanism of pm-Si:H deposition, as this signal ratio provides an indication of the probability of nanocrystal formation, as this process requires the polymerization of the silane species. Ifuku *et al*. have experimentally studied the behavior of plasma generated nanocrystals under different relative H_2_ flow ratios and have shown that the average particle size decreases when SiH_4_ was diluted by H_2_^28^. The nucleation and agglomeration of the species or particles are also enhanced by a larger concentration of precursors (either SiH_4_ partial pressure or total pressure). In other words, deposition rate (r_d_) of the film also depends on gas flow ratio R. At high gas flow ratio, relatively high SiH4 partial pressure results in higher concentration of nanocrystals, and it will lead to higher r_d_. Figure 2(c) shows that the r_d_ increases with SiH_4_ flow rate. This is now an interesting result because the increase in r_d_ is also correlated with the increase in Si_2_H_x_ and the Si_3_H_x_ concentration, which also increase with higher SiH_4_ flow rate. It suggests that the high r_d_ of pm-Si:H occurs simultaneously with the growth of silicon nanocrystals in the plasma. As previously indicated, a low SiH_4_ flow rate (highly diluted silane gas) leads to μc-Si:H deposition. In this region, the low Si_2_H_x_ intensity can be attributed to the fact that the increase in dilution (H_2_) favors the reverse reaction of formation of silicon clusters^26^:


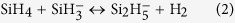


Moreover, the increase in hydrogen dilution may also increase the concentration of atomic H which can also prevent agglomeration of growth precursors by passivating the surface of nanocrystals. It is important to consider why a-Si:H deposition conditions also result in a low concentration of higher order silane species. Indeed, a-Si:H deposition is performed at low pressure (45 mTorr) from the dissociation of pure SiH_4_ (50 sccm) at low RF power of 1 W. Under these conditions, nanocrystal formation is suppressed as it strongly depends on SiH_4_ partial pressure^28^,^29^. Therefore, formation of higher order silane species is not expected in a-Si:H deposition conditions. On the other hand, it should be also pointed out that although the deposition condition of a-Si:H at R = 1 suppresses nucleation, it also leads to low deposition rate as shown in Fig. 2(c).

The data of Figs 1 and 2 can be interpreted with reference to the formation of nanocrystals and powders in the plasma. It has been widely studied that powder formation in the plasma is initiated by the nucleation and agglomeration of particles (less than 3 nm in diameter), and begins through polymerization of the silane species^30^. Powder formation is indeed very pronounced under high pressure, and pm-Si:H is produced under plasma conditions close to powder formation. The most significant differences in plasma conditions between pm-Si:H and a-Si:H are in the higher pressure and higher RF power^16^, which increase the residence time and the electron density, respectively. The presence of primary radicals and longer residence times increases the probability of collisions between radicals (favors the forward reaction in Eq. 2). Primary negative ions can react with parent gas molecules, giving rise to a first nucleation, but additional polymerization reactions need a hydrogen loss from the surface. However, negative ions, being confined in the plasma, can strongly polymerize in a chain reaction and contribute to growth of the particles inside the plasma^25^,^26^. The increase in the total pressure results in an increase in the density of growth precursors in the plasma, followed by the onset of agglomeration along with a sharp increase in the r_d_. Such transition to the “dusty” regime in silane plasma chemistry is called the α–γ′transition^31^,^32^.

**Figure 3:**
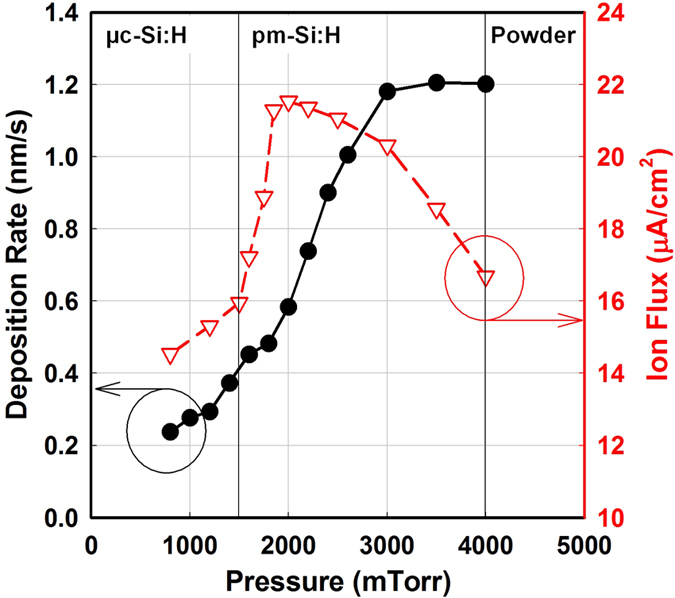
Deposition rate and ion flux as functions of the deposition pressure. At low pressure (pristine plasma) the deposited films are microcrystalline. The pm-Si:H conditions corresponds to the sharp rise in the ion flux. Above 3 Torr the decrease in ion flux is related to the formation of large clusters and powder which deteriorate the film quality. The pressure series were deposited at substrate temperature of 200 °C, RF power density of 75 mW/cm^2^, SiH_4_ and H_2_ gas flow of 30 and 400 sccm, respectively. Interelectrode distance was set to be 12 mm.

Figure 3 shows the deposition rate (r_d_) and the ion flux to the substrate as functions of the process pressure. The ion flux was measured by a plasma impedance probe installed between the matching box and the RF electrode. In Fig. 3, one can distinguish three regions regarding the flux of reactive species. At low pressure, both deposition rate and ion flux show a moderate increase as a function of the process pressure. Above 1.75 Torr, the deposition rate shows a more rapid increase, as does the ion flux. The ion flux reaches a plateau of ~20 μA/cm^2^ at around 2 Torr after its sudden jump, while the deposition rate shows a continuous increase. This can be interpreted as an increase in the plasma density as the injected power is coupled more efficiently to electrons, and which will result in more growth species both for film growth and nanocrystal formation. At pressures above 3 Torr, the deposition rate no longer increases, while the ion flux shows a fast decrease. We understand that formation of powder gets more significant at such high pressure, and those powders grow too large so that they are negatively charged, and decrease the electron density in the plasma. Those large powders would get flushed to the pumping line through the gas flow drift^28^. Thus, above 3 Torr there is a decrease in the ion flux due to the lower plasma density (Fig. 3), as well as in the ion impact energy as the sheath becomes highly collisional^12^,^33^. In addition, high pressure deposition conditions such as above 4 Torr would risk powder formation that is associated with a deterioration of the film quality due to the production of pinholes, roughness, porosity, and columnar growth^34^,^35^,^36^.

**Figure 4:**
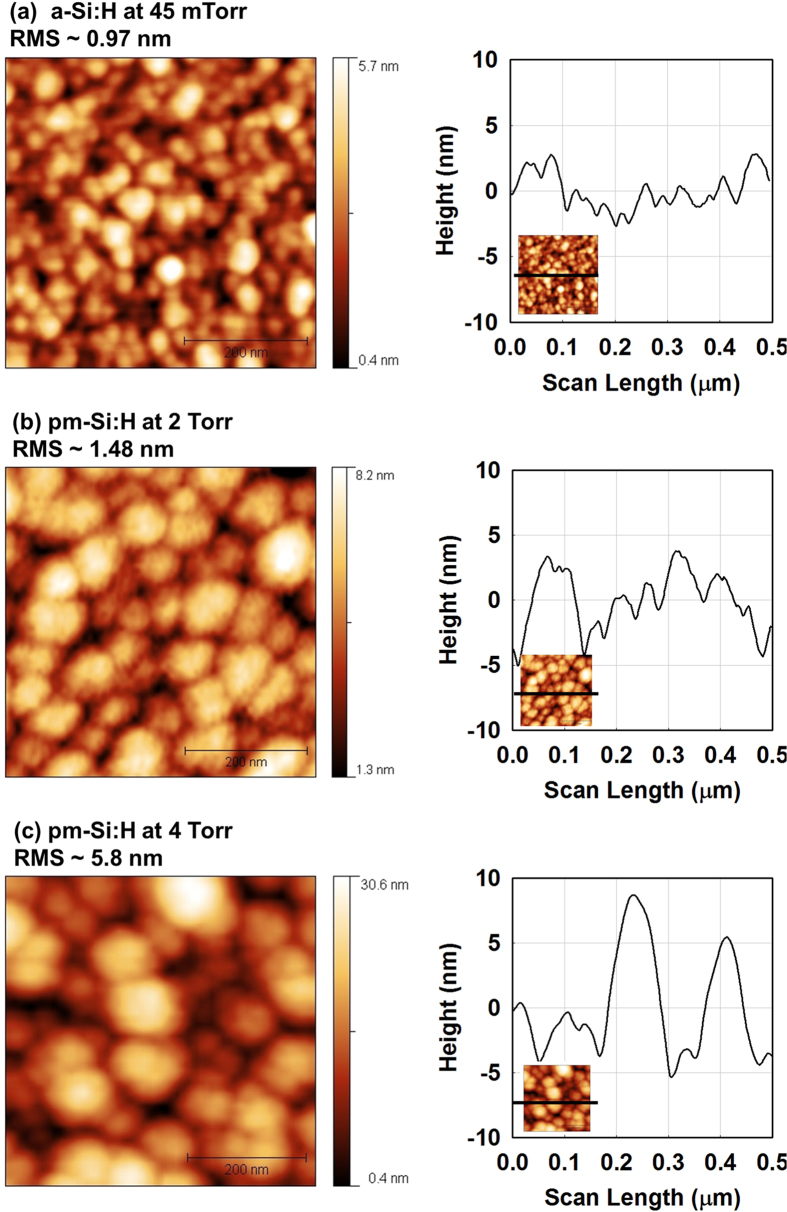
AFM images and surface profiles of (**a**) standard a-Si:H, (**b**) pm-Si:H deposited at 2 Torr, and (**c**) pm-Si:H deposited at 4 Torr. Note that at higher pressure, larger surface features and rougher surfaces are observed. Also, the size of the bumps cannot be attributed to individual nanoparticles (a few nanometers).

Figure 4 shows atomic force microscopy (AFM) images of a standard a-Si:H film and these of pm-Si:H films deposited at 2 Torr and 4 Torr. Increasing the pressure from 45 mTorr to 4 Torr leads to an increase in both RMS roughness and surface feature size. The surface features in Fig. 4(b) and (c) can be interpreted as surface roughness introduced by high sticking coefficient of silicon clusters and nanocrystals. It should be noted that the height of the surface features (several nanometers) is about one order of magnitude smaller than that of their lateral dimensions (about hundred nanometers). The deposition at pressure above 3 Torr results in material with high roughness and high porosity.

**Figure 5:**
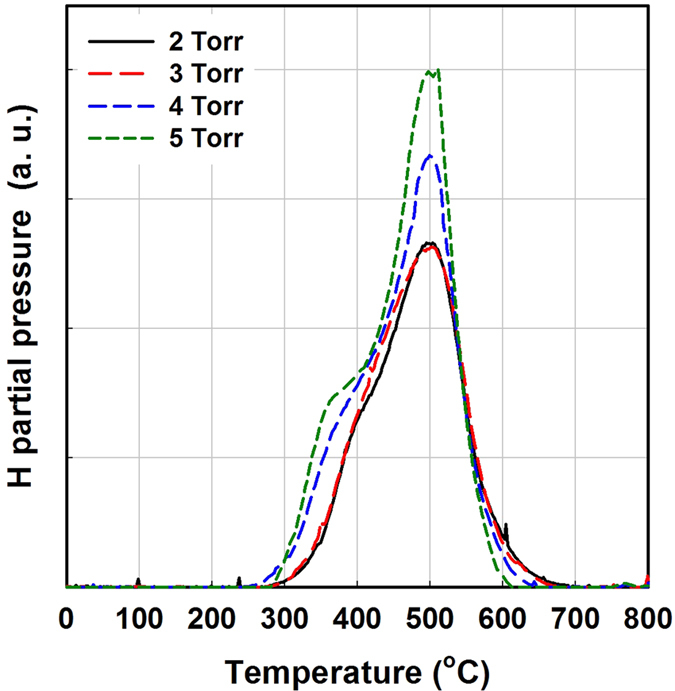
Hydrogen exodiffusion spectra of a set of pm-Si:H films deposited under different pressures, from 2 to 5 Torr.

Hydrogen exodiffusion is a powerful technique to detect hydrogen bonding configurations (related to the film microstructure) and to detect interconnected voids in the material, which for a-Si:H materials have been shown to be a major source of defects^37^,^38^. Figure 5 shows the hydrogen exodiffusion spectra of a set of pm-Si:H films deposited under different pressure values in the range from 2 to 5 Torr. The spectra are normalized to the volume of the film. The hydrogen exodiffusion result shows that increasing deposition pressure increases hydrogen evolution at both 350 °C and 500 °C. In particular, hydrogen evolution at 350 °C is closely related to the porosity of the material (weakly bonded hydrogen). A material with a high degree of columnar morphology or extensive internal inhomogeneity will lead to hydrogen evolution between 300–400 °C, with further evolution near 500–600 °C. On the contrary, compact films are characterized by a single peak at high temperature (500 °C)^39^. The peak at 300–400 °C is also thought to originate from molecular hydrogen release from the internal surfaces of interconnected voids^40^ or from silicon nanocrystal surfaces^41^. Moreover, in case of silicon nanocrystals, the low temperature hydrogen effusion peak shifts to lower temperature when larger nanocrystals are incorporated^41^. Another interesting point is that the shoulder of exodiffusion spectra at 600 °C slightly decreases at higher deposition pressure. The exodiffusion shoulder at 600 °C has been attributed to hydrogen evolution from isolated voids^42^. Therefore, the increase in deposition pressure of pm-Si:H results in a decrease of the isolated void density and increase in interconnected void density. Such trend could also imply a greater internal surface area, providing more space for hydrogen. Moreover, high pressure deposition also increases the hydrogen evolution signal at 500 °C, which is associated to tightly bonded hydrogen^39^. The existence of a low temperature exodiffusion peak can be also interpreted as incomplete structural re-construction^37^.

The above results indicate that the denser material is deposited under plasma conditions immediately after the transition to pm-Si:H, such as a pressure around 2–3 Torr and R value between 0.05 to 0.1. Interestingly, this corresponds to high deposition rate (Fig. 3) compatible with a dense network (Fig. 5) and is related to a high flux of positively charged species (Fig. 3). As a matter of fact, the drop in ion flux above 3 Torr does not affect the deposition rate but results in the formation of a more porous material, most likely related to the formation of large clusters and powder as it has also been reported for hydrogenated polymorphous silicon carbon films^43^.

Our results so far show that process conditions for pm-Si:H result in the formation of higher silanes which are the precursors for the formation of nanocrystals. Their density increases with the total pressure until they form agglomerates up to few nanometers. This process can continue by the formation of larger agglomerates and ends with the formation of larger powders. We now consider if the nanocrystals with diameters less than 5 nm can contribute significantly to the film growth^44^. Depending on the plasma conditions, the whole process from primary particle nucleation to powder formation can take a few seconds, or it can be sustained at a steady-state at some intermediate stage. In the case of pm-Si:H deposition, the agglomeration phase is avoided by keeping the pressure at values for which the characteristic time for diffusion to the substrate is smaller than the agglomeration time^45^.

**Figure 6:**
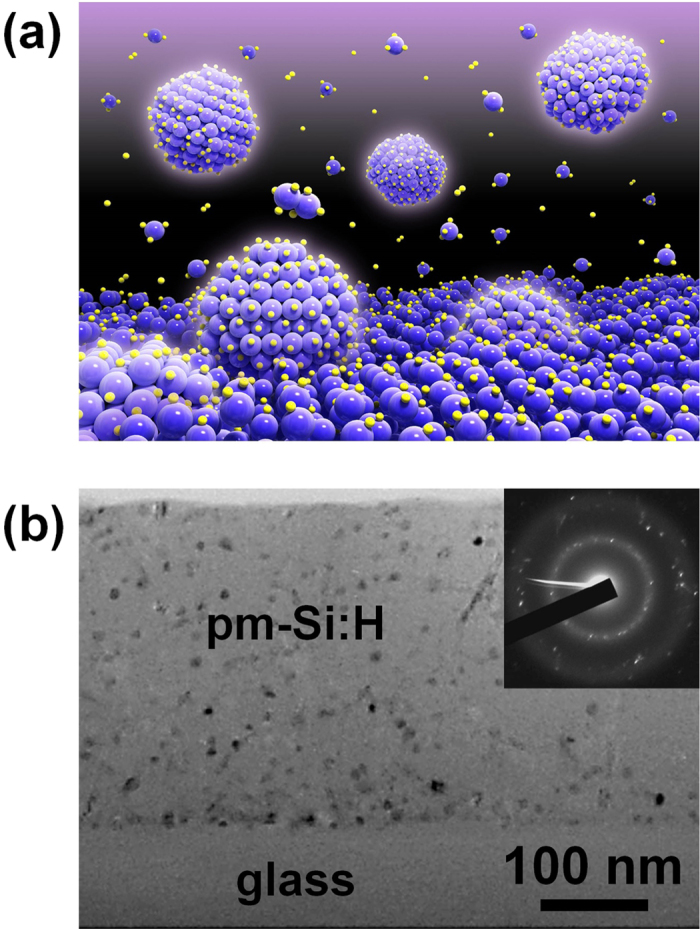
(**a**) Schematic representation of the pm-Si:H deposition process, mainly regarding plasma synthesized silicon nanocrystals contributing to film deposition. In contrast with a-Si:H deposition process which relies on the formation and surface reaction probability of SiH_3_ radicals, pm-Si:H deposition is mostly based on the contribution of silicon nanocrystals synthesized in the plasma. (**b**) Cross-section TEM image of 300 nm thick pm-Si:H film deposited on glass substrate.

**Figure 7:**
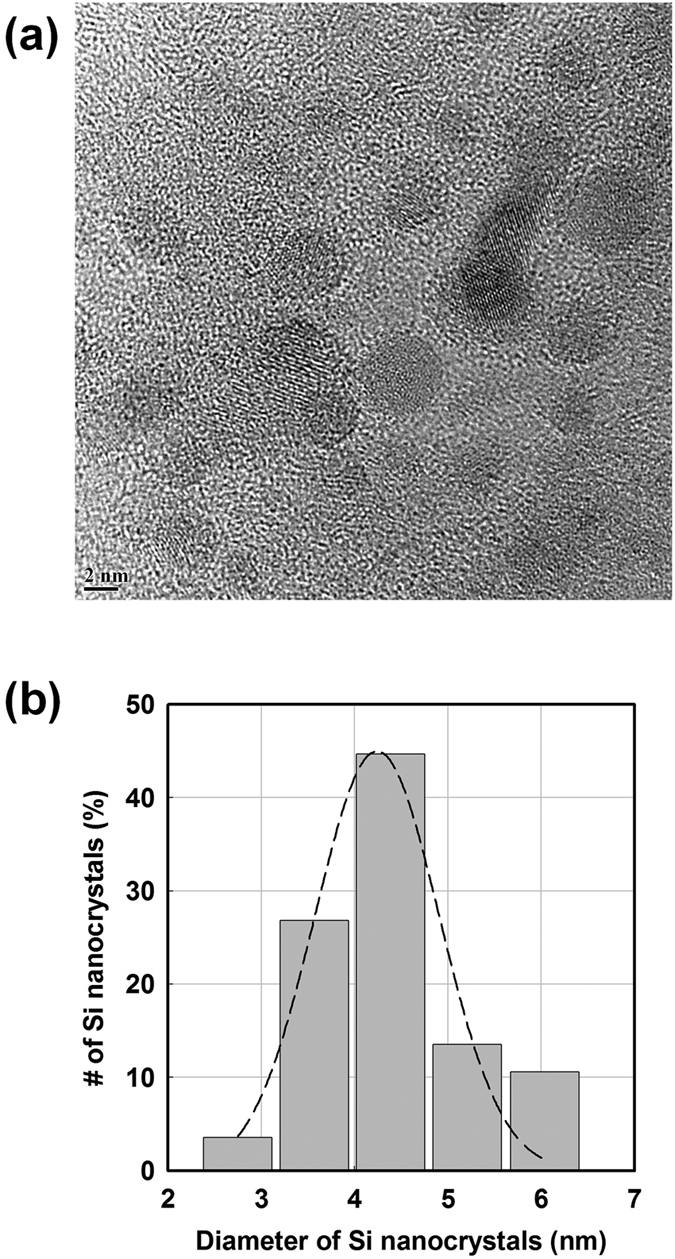
(**a**) HRTEM image of a pm-Si:H film deposited at 2 Torr, and (**b**) the size distribution of silicon nanocrystals obtained from the HRTEM in (**a**). The average diameter of the nanocrystals in the sample is found to be 4.2 ± 1.0 nm.

The contribution of nanometer-size nanocrystals along with that of silicon radials to deposition is illustrated Fig. 6(a) which is a schematic representation of the pm-Si:H deposition process. This view is fully supported by the transmission electron microscopy (TEM) image in Fig. 6(b) showing the cross-section of a 300 nm thick pm-Si:H film deposited at 5 Torr on a glass substrate. One can find a homogeneous distribution of silicon nanocrystals throughout the whole film thickness, indicating that the process of silicon nanocrystal formation and deposition can be sustained in a steady-state condition at such pressure, without turning into the dust phase. As discussed in the introduction, we conjecture that the pm-Si:H growth process to result from the direct incorporation of silicon nanocrystals synthesized in the plasma and not from the nucleation of crystallites in the film, which is the feature that defines protocrystalline and then microcrystalline growth. The fact that silicon nanocrystals are homogeneously dispersed through the film thickness further supports our hypothesis of pm-Si:H deposition resulting from the contribution of silicon nanocrystals formed in the gas phase, as does their spherical shape. Usually PECVD grown μc-Si:H films based on SiH_3_ radicals show characteristic grain growth in conical shape that crystalline growth on amorphous incubation layer and growth from many small nanocrystal seeds into few larger ones^20^,^46^. However, the microstructure of pm-Si:H revealed in our TEM cross-section shows spherical shape indicating that the origin of silicon nanocrystals is the plasma synthesize in gas phase. At this pressure, the size distribution of the nanocrystals is in the range of 5–10 nm and their crystalline nature is demonstrated by the electron diffraction pattern shown in the inset in Fig. 6(b). Finally, Fig. 7(a) shows a high resolution TEM (HRTEM) image (top-view of film) of a sample deposited at 2 Torr. One can also notice the spherical shape of the nanocrystals, supporting the hypothesis that they were produced in the plasma. Their size distribution, obtained from the analysis of the HRTEM image is presented in the histogram of Fig. 7(b). At this pressure, the average diameter of nanocrystals is found to be 4.2 ± 1.0 nm; i.e. smaller than for the sample deposited at 5 Torr. It should be noted that for these films, the crystalline fraction measured by Raman and by SE is very small (<10%), and only by X-ray diffraction or TEM can a crystalline fraction be unambiguously observed.

## Conclusions

The growth mechanism and structure of hydrogenated polymorphous silicon thin films have been studied with respect to the deposition conditions. High resolution TEM studies of the film cross-section provide experimental evidence for the continuous contribution of silicon nanocrystals to the film growth. These results are supported by QMS analysis and ion flux measurements which support our previous results on the best pm-Si:H material being obtained under process conditions after the transition from a pristine plasma to a plasma containing nanocrystals, such as pressure around 2–3 Torr and R values between 0.05 to 0.1 which also coincide with the highest deposition rate achievable deposition for fixed values of the RF power and silane flow rate. Even though higher pressure and larger SiH_4_ flow result in high deposition rate, such process conditions risk poor material due to powder formation. These results are of crucial importance for further optimization of optoelectronic devices based on pm-Si:H films.

## Methods

Hydrogenated polymorphous silicon thin films were deposited by the capacitively-coupled-plasma (CCP) radio-frequency (RF, 13.56 MHz) glow discharge PECVD method at substrate temperatures ranging from 175 to 200 °C. Two PECVD systems (one in France and the other in South Korea) were used. The tool in France has a multiplasma-monochamber configuration^47^ while the one in South Korea is a cluster tool consisting of five independent PECVD reactors. Despite the different designs and sizes of the two PECVD systems, we have obtained very similar plasma behavior for common deposition process conditions (for example, the pressure at the alpha-gamma transition). Intrinsic pm-Si:H films were deposited under carefully controlled plasma conditions using hydrogen-diluted silane gas mixtures. In this work, our pm-Si:H layers were deposited at pressure range from 1.5 to 5 Torr and RF power density in a range from 30 mW/cm^2^ to 100 mW/cm^2^ using inter electrode distance of 12 mm. Standard a-Si:H, used as a control, was obtained by the dissociation of pure silane at low pressure (50 mTorr) and low RF power density (5 mW/cm^2^) with inter electrode distance of 28 mm.

The formation of higher order silane molecules (the precursor for nanocrystal formation) during plasma processing was monitored by quadrupole mass spectrometry (QMS) and their effect on the electrical properties of the discharge via a plasma impedance probe. The QMS is an Extorr 100, which consists of an ionizer, quadrupole mass filter and detector, and it was installed in the pumping line, right after the throttle valve, in order to scan the molecular mass distribution of plasma species during deposition. For ion flux measurements, an Octiv Suite VI Probe was installed between the matching box and the RF electrode in order to use the RF electrode as a sensor. This probe extracts the ion saturation current from the RF current and voltage curves.

To characterize thin film thickness and material composition, spectroscopic ellipsometry (SE) was performed on the films deposited on glass substrates. Note that the same process conditions on <100> c-Si substrates have been shown to lead to epitaxial growth^48^. The SE spectra were modeled using the Tauc-Lorentz dispersion model for amorphous materials^49^,^50^, and the Bruggeman effective medium approximation (BEMA) was used to determine the composition of μc-Si:H films^51^,^52^.

The surface morphology of the films was characterized by atomic force microscopy (AFM). Tapping mode was used to prevent the cantilever from dragging across the surface and resulting in surface damage, while providing high resolution. The scan rate was chosen to be slow, from 1 to 2 Hz, because the surface of the films displays low roughness and small features. Scanning area size was varied from 500 × 500 nm^2^ to 2 × 2 μm^2^. Sets of AFM images were analyzed by surface grain extraction, from which the surface feature size and distribution were obtained.

Hydrogen exodiffusion experiments were performed in order to characterize the hydrogen-related microstructure of the films, in particular the presence of interconnected voids, which is a major source of defects in silicon thin film. During hydrogen exodiffusion experiments, the base vacuum was 10^−7^ mbar and the heating rate was 10 °C/min. The effused hydrogen was detected by a QMS, and recorded in a continuous manner with the increase in temperature, to obtain the hydrogen effusion spectrum. Finally, the structure of the films was studied by transmission electron microscopy (TEM) on a JEM-2100F transmission electron microscope (JEOL) at an accelerating voltage of 200 kV. In particular, a cross-section of the sample was prepared for TEM observation in order to characterize the distribution of nanocrystals across the film thickness.

## Additional Information

**How to cite this article**: Kim, K.-H. *et al*. Unravelling a simple method for the low temperature synthesis of silicon nanocrystals and monolithic nanocrystalline thin films. *Sci. Rep.* **7**, 40553; doi: 10.1038/srep40553 (2017).

**Publisher's note:** Springer Nature remains neutral with regard to jurisdictional claims in published maps and institutional affiliations.
